# Mediating effects of sense of coherence and psychological resilience on stigma and quality of life among postoperative middle-aged and older patients with malignant gynecological tumors: a cross-sectional, structural equation model

**DOI:** 10.1007/s00520-025-09818-7

**Published:** 2025-08-16

**Authors:** Pinying Chen, Shaoyuan Xu, Jinxia Nian, Yuqing Pan, Xiumin Jiang

**Affiliations:** 1 School of Nursing, Fujian Medical University, Fuzhou City, Fujian Province China; 2 Fujian Maternity and Child Health Hospital College of Clinical Medicine for Obstetrics & Gynecology and Pediatrics, Fujian Medical University, Fuzhou City, Fujian Province China

**Keywords:** Malignant gynecological tumor, Quality of life, Stigma, Psychological resilience, Sense of coherence

## Abstract

**Purpose:**

Middle-aged and older individuals are at a crucial stage of aging, marked by increased vulnerability to psychological and physiological challenges. Cancer has a negative impact on the quality of life of patients. Stigma is closely related to the quality of life (QoL) of middle-aged and older patients with malignant gynecological tumors, but the mechanism behind this relationship is still unclear. Sense of coherence (SOC) and psychological resilience (PR) are positive mental health factors that can alleviate psychological stress and improve the quality of life. Therefore, this study aimed to explore the mediating role of sense of coherence and psychological resilience between stigma and quality of life.

**Methods:**

A cross-sectional survey was conducted from May 2023 to January 2024, involving a total of 428 postoperative patients from the gynecological oncology departments of four tertiary grade A hospitals in Fujian Province. Assessment tools included the Functional Assessment of Cancer Therapy-General scale, the Social Impact Scale, the Connor–Davidson Resilience Scale, and the Sense of Coherence Scale-13. Data analysis was performed using IBM SPSS 26.0 and AMOS 24.0 software.

**Results:**

Descriptive analyses showed generally low quality of life levels. Stigma negatively affected psychological resilience and quality of life, while psychological resilience positively affected quality of life. Stigma also negatively influenced the sense of coherence, whereas the sense of coherence positively affected quality of life. Furthermore, the sense of coherence had a positive effect on psychological resilience. The sense of coherence and psychological resilience partially mediated the relationship between stigma and quality of life.

**Conclusions:**

Stigma has a direct impact on the quality of life and also exerts an indirect effect through the mediation of sense of coherence and psychological resilience. Clinical healthcare providers can improve patients’ quality of life by reducing stigma and promoting sense of coherence and psychological resilience.

**Supplementary Information:**

The online version contains supplementary material available at 10.1007/s00520-025-09818-7.

## Introduction

Malignant gynecological tumors are one of the most common cancers affecting women. According to the International Agency for Research on Cancer [1], approximately 1.47million new cases of malignant gynecological tumors were reported in 2022, representing 7.30% of all cancer cases globally, with around 679,549 deaths, accounting for 7.00% of the global cancer mortality. The annual incidence of these tumors continues to rise, with cervical, endometrial, and ovarian cancers being the most prevalent. These cancers pose significant threats to women’s health and well-being, particularly among middle-aged and older adults who are at higher risk. The incidence and mortality of cervical cancer increase with age, peaking between the ages of 50 and 54. The average age at diagnosis is 53 years and the average age at death is 59 years, though variations exist across countries and regions [2]. Additionally, about 50% of ovarian cancer cases occur in women over 65 [3] and the average age at diagnosis for endometrial cancer is 61 years [4].

Due to the importance of reproductive organs such as the uterus and ovaries, women with malignant gynecological tumors often experience greater mental stress and psychological challenges compared to patients with other types of cancer. These challenges can severely impact their physical and mental health, weaken their social support systems, affect treatment outcomes, and significantly diminish their quality of life (QoL) [5]. Previous studies have shown that malignant gynecological tumors profoundly affect both physical and psychological well-being [6]. Given that middle-aged and older adults are considered a vulnerable group in society, they also face high health risks. These individuals are either in or about to enter the critical period of aging. Compared with younger individuals, their physical function is declining and they are prone to functional decline, which impacts their QoL [7]. Including this group in QoL research offers a new perspective in the field and holds valuable implications for the development and enhancement of relevant policies aimed at improving the QoL and overall well-being of middle-aged and older patients with malignant gynecological tumors.

The World Health Organization defined QoL as “an individual’s subjective evaluation of their role and status in life, based on their cultural background and values, as well as their personal goals, expectations, standards, and concerns.” This perspective emphasizes a subjective assessment within cultural, social, and environmental contexts, encompassing four key domains: physical health, psychological well-being, social relations, and the environmental factors [8]. QoL represents a comprehensive evaluation of a patient’s physical health, mental health, and social adaptability [9]. Previous studies indicated that the QoL of cancer patients may be influenced by several factors, including education level, family support, income, sleep quality, smoking habits, tumor differentiation, and tumor size [10–12].

Stigma refers to the situation in which an individual is deprived of social recognition, resulting in being labeled and marked as different, leading to devaluation and denigration by others. It is a pervasive and negative social phenomenon characterized by stereotypes [13]. Previous studies have shown that stigma is a common psychological issue among patients with malignant tumors [14]. Stigma not only hinders disease treatment but also adversely affects patients’ physical and mental health, family and social relations, and most notably their QoL [15]. A negative correlation has been found between stigma and QoL, meaning that the higher the level of stigma, the lower the patient’s QoL [16]. Most existing research on the relationship between stigma and QoL in cancer patients has focused on breast cancer, rectal cancer, oral cancer, and lung cancer [15, 17–19]. However, there is limited research on middle-aged and older patients with malignant gynecological tumors, highlighting the need for further investigation in this area.

Resilience, as known as psychological resilience (PR), refers to the positive adaptive capacity of individuals or groups positive when facing stress, challenges, adversity, or trauma [20]. In this study, resilience specifically refers to the coping and adaptability of middle-aged and older patients with malignant gynecological tumors when confronting negative events such as disease and stress. Patients with malignant tumors often encounter challenges like negative emotions, fear of cancer recurrence, sexual dysfunction, and lack of social support during treatment [21]. Those with high resilience are better able to maintain a positive mental state and a higher QoL [22].

Sense of coherence (SOC) is defined as a universal, enduring, and dynamic confidence in one’s ability to manage internal and external environmental stressors [23]. It reflects an individual’s overall perception and cognitive approach to life, including their understanding of stress, their ability to select appropriate resources to respond to stressors, and their emotional valuation of facing stress [24]. Previous studies have identified SOC as a key indicator of mental health, with a significant positive impact on QoL. The mediating role of SOC between psychological factors such as negative emotions, coping styles, and QoL was further studied. Patients with higher SOC levels are better equipped to manage and enhance their QoL [25]. Previous studies indicated that PR can effectively improve patients’ QoL and play a regulatory role in stigma and QoL [26]. SOC, as an internal factor, influences the development of PR. Furthermore, some studies have found that higher levels of SOC are associated with better PR development [27].

**Fig. 1:**
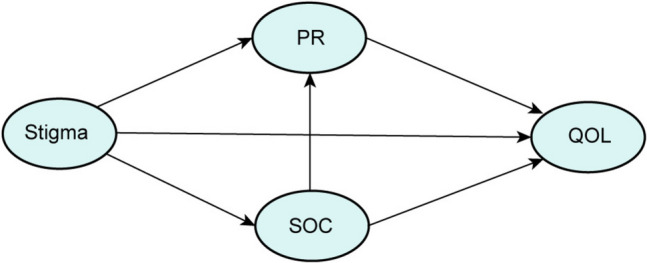
Theoretical model and hypotheses

This study is guided by the cognitive-affective-behavioral model [28] and resilience framework [29], which holds significant implications for the quality of life among patients. In accordance with this model, the interplay of individual cognition, emotion, behavior, and self-evaluation may lead to cognitive bias and a range of negative emotions, such as anxiety, depression, and shame, in middle-aged and elderly patients with gynecological malignant tumors. By leveraging the role of positive psychology, the original cognition is gradually transformed, thereby enhancing the quality of life. Based on the theoretical framework and literature review, the research hypotheses illustrated in Fig. 1 were formulated.

- Hypothesis 1: Stigma has a direct negative effect on quality of life.
- Hypothesis 2: SOC mediates the relationship between stigma and QoL.
- Hypothesis 3: PR mediates the relationship between stigma and QoL.
- Hypothesis 4: SOC and PR have a chain-mediating effect between stigma and QoL.

## Methods

### Study design and participants

A multi-hospital cross-sectional survey was conducted from May 2023 to January 2024, including 428 females from the gynecological departments of four tertiary grade A hospitals in Fujian Province, southeast China, using a convenient sampling method. The inclusion criteria were as follows: female patients who (1) were diagnosed with malignant gynecological tumors via pathological examination, (2) were aged between 45 and 74 years, (3) were first diagnosed and hospitalized post-surgery, (4) could communicate well in Chinese and independently complete the questionnaires, and (5) were willing to participate voluntarily. The exclusion criteria were as follows: (1) distant metastasis of cancer cells and (2) mental illness, cognitive impairment, or other severe life-threatening diseases.

### Sample size

QoL was the dependent variable in this study, and the sample size was calculated using the estimation method of sample size for current situation studies: *N* = (*u*_*α/2*_ × *σ/δ)*^*2*^ [30]. The probability level value was 0.05 and the standard deviation was 7.99, obtained from the pilot study analysis including 20 participants. The allowable error value was 0.80, which was 10% of the standard deviation, and the desired statistical power level was 0.95. To account for potential invalid questionnaires, the sample size was increased by 10%, resulting in a minimum sample size of 426. The researchers conducted a thorough validation of the completeness and accuracy of the questionnaires. Any questionnaire with missing items was deemed invalid. Additionally, questionnaires with uniform answer options, obvious patterns in option selection, or more than 10% of items missing were excluded.

### Data collection

Data were collected using on-site surveys administered by trained investigators. All surveys were administered in paper format. The questionnaire survey was conducted in the stable period of patients after operation, avoiding the peak period of treatment. After obtaining signed informed consent, the investigators introduced the purpose of the study and the questionnaire completion process. Participants completed a comprehensive questionnaire that included a general information questionnaire, Functional Assessment of Cancer Therapy-General (FACT-G), Social Impact Scale (SIS), Connor–Davidson Resilience Scale (CD-RISC), and Sense of Coherence Scale-13 (SOC-13). General information was obtained through brief interviews, and clinical data were obtained from the hospital’s electronic medical record system. The participants were free to withdraw from the study at any time and for any reason. Of the 460 questionnaires distributed, 11 patients withdrew and 21 invalid questionnaires were excluded, resulting in 428 valid responses (an effective response rate of 93.04%).

## Instruments

### General information questionnaire

The general information questionnaire was designed by the researchers based on a literature review and expert consultations [31]. It included the following information: age, ethnicity, religion, marital status, number of children, residential location, education level, employment status, per capita monthly household income, payment pattern, smoking habits, family caregiver (parents, children, siblings, other relatives); concomitant diseases, family history of cancer, diagnosis, surgical procedures, preoperative adjuvant treatment, and cancer stage.

### Quality of life (QoL)

In this study, QoL was measured using the Chinese version of FACT-G [32]. The FACT-G, developed by Cella et al. [33], is a standardized tool used to assess the QoL of patients with cancer. It consists of 27 items (e.g., “I am compelled by my illness to stay in bed”) and encompasses four dimensions: physiological well-being (PWB, seven items), social/family well-being (SWB, seven items), emotional well-being (EWB, six items), and functional well-being (FWB, seven items). Each item is scored on a 5-point Likert scale ranging from 0 (not at all) to 4 (very much), with higher scores indicating better QoL. The total score ranges from 0 to 108. The Chinese version of the FACT-G has a test–retest reliability of over 0.85 across all four domains, with Cronbach’s alpha coefficients all exceeding 0.80. The content validity index is 0.927, indicating good reliability and validity. Cronbach’s *α* value for this scale was 0.885 in this study.

### Stigma

The 24-item SIS, translated and adapted for Chinese by Pan et al. [34], is commonly used to assess stigma in individuals with chronic illnesses, disabilities, and cancers [35]. It evaluates four dimensions of perceived stigma: financial insecurity (FI, three items), social rejection (SR, nine items), internalized shame (IS, five items), and social isolation (SI, seven items). Each item is scored on a 4-point Likert scale ranging from 1 (strongly disagree) to 4 (strongly agree). The total score ranges from 24 to 96, with higher scores indicating greater stigma. A previous study validated the Chinese version of the SIS, with a separation reliability of 0.99 [36]. Cronbach’s *α* value for this scale was 0.901 in this study.

### Psychological resilience (PR)

The CD-RISC developed by Conner and Davidson [37] and translated into Chinese by Yu X. N. and Zhang J. X. [38] was used to assess PR. The CD-RISC is a reliable tool for measuring an individual’s PR and has been widely applied to cancer patients [39]. The Chinese vision of the CD-RISC, tested in cancer patients, demonstrates good construct validity, with Cronbach’s *α* coefficient of 0.952 and a content validity index of 0.91 [39]. The scale consists of 25 items divided into three dimensions: resilience (13 items), strength (eight items), and optimism (four items). Each item is scored on a 5-point Likert, with higher scores reflecting greater resilience. Cronbach’s *α* value for this scale was 0.942 in this study.

### Sense of coherence (SOC)

SOC was measured using the Chinese version of the SOC-13 [40], which is a concise version of the original 29-item scale [24]. This scale includes 13 items aggregated into three components: comprehensibility (CB), manageability (MB), and meaningfulness (MF). The total SOC score was calculated by summing the included items, with certain items reverse-scored. The overall score ranges from 13 to 91, with higher scores indicating a stronger SOC. The Chinese version of the SOC-13 has good internal consistency, with Cronbach’s alpha of 0.87 [41], and has been widely used in cancer studies [42]. Cronbach’s *α* value for this scale was 0.887 in this study.

### Statistical analyses

Statistical analyses were performed using SPSS (version 26.0) and AMOS (version 24.0) software. All data were normally distributed. Descriptive analysis, Student’s *t*-test, and one-way analysis of variance (ANOVA) were used to analyze the mean QoL scores across different characteristic categories. Pearson’s correlation analysis was used to assess the relationships between the variables. Multiple linear regression models were constructed with QoL as the dependent variable, and stigma, PR, and SOC as independent variables, controlling for all characteristic variables. Structural equation modeling (SEM) identified the mediating effects of SOC and PR between stigma and QoL. All tests were two-sided, and a *p*-value of < 0.05 was regarded as statistically significant. The hypothetical model’s fitness was evaluated using the following thresholds: chi-square/degrees of freedom ratio (x^2^*/df* < 5.00), goodness-of-fit index (GFI ≥ 0.80), adjusted goodness-of-fit index (AGFI > 0.80), normed fit index (NFI ≥ 0.90), incremental fit index (IFI ≥ 0.90), Tucker–Lewis fit index (TLI ≥ 0.90), comparative fit index (CFI > 0.90), and root mean square error of approximation (RMSEA ≤ 0.08) [43]. The significance of the indirect and total effects of the model was tested using the bootstrap method with 5000 bootstrapping iterations.

## Results

### Demographic characteristics

Table 1 presents the participants’ demographic characteristics. A total of 460 females participated in this study’s survey, with 428 valid responses (response rate: 93.04%). The average age of the participants was 56.95 ± 7.99 years, with the majority (65.00%) aged between 45 and 59 years old. Most participants (98.1%) were of Han ethnicity and 24.5% had religious beliefs, including Buddhism, Taoism, and Christianity. Most participants were married (93.9%), 94.8% had one or more children, and 38.8% resided in rural areas. Additionally, 81.5% had a junior high school education or below, 61.2% were currently unemployed, and the average monthly household income was predominantly < 3000 CNY (46.5%). Furthermore, most participants had medical insurance (97.9%) and were non-smokers (98.8%). In addition, 71.5% primarily relied on their spouses as caregivers and 64.72% had concomitant diseases. Additionally, 46.96%, 26.4%, and 21.0% of participants were diagnosed with cervical, endometrial, and ovarian cancers, respectively. Finally, approximately 54.9% were in cancer stage I, and the primary surgical approach was unreserved fertility, representing 95.8% of cases.

**Table 1:** Distribution of characteristics and univariate analyses of quality of life (*n* = 428)

Characteristics	Categories	N	%	Mean ± SD^4^	*t*/*F*	p
Age (years)	45–59	278	65.0	62.35 ± 12.07	4.496	< 0.001^**^
	60–74	150	35.0	56.99 ± 11.17		
Ethnicity	Han	420	98.1	60.39 ± 12.09	− 1.015	0.311
	Others	8	1.9	64.75 ± 7.13		
Religion	Yes	105	24.5	63.01 ± 11.19	− 2.502	0.013^*^
	No	323	75.5	59.65 ± 12.19		
Marital status	Unmarried	5	1.2	56.40 ± 16.55	0.310	0.818
	Married	402	93.9	60.51 ± 12.11		
	Divorced	11	2.6	62.27 ± 9.76		
	Widowed	10	2.3	59.20 ± 9.11		
Number of children	None	22	5.1	60.27 ± 12.05	5.166	0.002^**^
	1	141	33.0	63.51 ± 10.94		
	2	155	36.2	59.61 ± 12.21		
	≥ 3	110	25.7	57.85 ± 12.42		
Residential location	Rural	166	38.8	59.60 ± 12.28	2.140	0.119
	Towns	102	23.8	59.46 ± 10.90		
	Urban	160	37.4	62.03 ± 12.36		
Education level	Junior school and below	349	81.5	59.48 ± 12.00	4.931	0.002^**^
	High school or technical secondary school	45	10.5	63.47 ± 12.04		
	Junior college	20	4.7	67.45 ± 7.94		
	Bachelor’s degree and above	14	3.3	65.57 ± 12.46		
Employment status	Employed	93	21.7	63.48 ± 11.64	5.639	0.004^**^
	Not employed	262	61.2	58.98 ± 11.68		
	Retired	73	17.1	62.00 ± 12.97		
Per capita monthly household income	< 3000 CNY^1^	199	46.5	58.75 ± 11.89	5.070	0.002^**^
	3000–5999 CNY	187	43.7	61.10 ± 11.87		
	6000–8999 CNY	32	7.5	67.25 ± 12.61		
	≥ 9000 CNY	10	2.3	61.30 ± 7.60		
Payment pattern	Insurance	419	97.9	60.49 ± 12.11	− 0.335	0.745
	Self-paying	9	2.1	59.67 ± 7.18		
Smoking habits	Yes	5	1.2	56.20 ± 9.26	− 0.799	0.425
	No	423	98.8	60.52 ± 12.05		
Family caregiver	Spouse	306	71.5	61.32 ± 11.85	3.503	0.008^**^
	Parents	8	1.9	66.25 ± 9.88		
	Children	101	23.6	56.92 ± 11.66		
	Siblings	8	1.9	64.88 ± 13.61		
	Other relatives	5	1.1	64.00 ± 18.37		
Concomitant diseases^2^	Yes	277	64.7	59.57 ± 11.58	2.123	0.034^*^
	No	151	35.3	62.14 ± 12.68		
Family history of cancer	Yes	101	23.6	59.41 ± 12.04	1.021	0.308
	No	327	76.4	60.80 ± 12.02		
Diagnosis	Cervical cancer	201	47.0	59.91 ± 12.49	4.157	0.008^**^
	Endometrial cancer	113	26.4	62.77 ± 10.64		
	Ovarian cancer	90	21.0	60.12 ± 12.95		
	Others^3^	24	5.6	55.75 ± 8.82		
Surgical procedures	Preservative fertility	18	4.2	64.39 ± 14.00	1.413	0.159
	Unreserved fertility	410	95.8	60.30 ± 11.92		
Preoperative adjuvant treatment	No	395	92.3	61.39 ± 11.86	8.007	< 0.001^**^
	Chemotherapy/radiotherapy	33	7.7	49.45 ± 7.85		
Cancer stage	Stage I	235	54.9	64.89 ± 10.03	45.851	< 0.001^**^
	Stage II	113	26.4	56.81 ± 11.86		
	Stage III	80	18.7	52.68 ± 12.06		

^1^CNY Chinese yuan (￥); ^2^concomitant diseases include hypertension, diabetes, asthma, and coronary heart disease; ^3^thers include vulvar cancer, vaginal cancer, fallopian tube cancer, choriocarcinoma, uterine sarcoma; ^4^standard deviation; ^*^p < 0.05; ^**^p < 0.01

### Univariate analysis of QoL among middle-aged and older patients with malignant gynecological tumors

The single-factor analysis results revealed significant differences in the factors affecting QoL. These factors included age, religion, number of children, education level, employment status, per capita monthly household income, family caregiver, presence of concomitant diseases, diagnosis, whether preoperative adjuvant treatment was performed, and cancer stage (Table 1, *p* < 0.05).

Descriptive statistics of measured variables: QoL, Stigma, PR, and SOC.

Table 2 presents the descriptive statistics of the measured variables. The average postoperative QoL score was 60.47 ± 12.03. The mean scores for stigma and SOC in this study were 65.41 ± 9.12 and 54.73 ± 10.55, respectively. Additionally, the mean score for PR was 55.39 ± 12.51, which is significantly lower than the national norm of 65.40 ± 13.90 (*t* =  − 16.550, *p* < 0.001) [44].

**Table 2:** Descriptive statistics of the measured variables (*n* = 428)

Variables	Mean ± SD	Min	Max	Skewness	Kurtosis
QoL	60.47 ± 12.03	26.00	95.00	− 0.097	− 0.373
PWB	16.55 ± 4.15	0.00	27.00	− 0.295	0.386
SWB	18.49 ± 4.20	3.00	28.00	− 0.482	0.176
EWB	14.76 ± 3.75	1.00	24.00	− 0.392	0.424
FWB	10.67 ± 4.25	0.00	28.00	0.459	1.287
Stigma	65.41 ± 9.12	27.00	94.00	− 0.685	1.906
FI	8.70 ± 1.57	3.00	12.00	− 0.092	1.265
SR	21.80 ± 3.58	9.00	34.00	− 0.235	1.840
IS	14.90 ± 2.59	5.00	20.00	− 0.463	0.853
SI	20.01 ± 3.35	7.00	28.00	− 0.593	1.307
PR	55.39 ± 12.51	25.00	97.00	0.747	1.163
Resilience	27.87 ± 7.05	11.00	52.00	0.690	1.074
Strength	18.72 ± 4.32	9.00	32.00	0.584	1.032
Optimism	8.80 ± 2.22	1.00	16.00	0.592	1.630
SOC	54.73 ± 10.55	33.00	90.00	0.338	− 0.322
CB	20.85 ± 4.28	12.00	35.00	0.227	− 0.378
MB	16.61 ± 3.84	7.00	28.00	0.786	0.182
MF	17.27 ± 3.63	8.00	28.00	0.280	− 0.093

*QoL*, quality of life; *PWB*, physiological well-being; *SWB*, social/family well-being; *EWB*, emotional well-being; *FWB*, functional well-being; *FI*, financial insecurity; *SR*, social rejection; *IS*, internalized shame; *SI*, social isolation; *PR*, psychological resilience; *SOC*, sense of coherence; *CB*, comprehensibility; *MB*, manageability; *MF*, meaningfulness

### Correlations between QoL, Stigma, PR, and SOC

Table 3 presents the Pearson correlation analysis results, indicating that QoL and its dimensions negatively correlated with stigma and its dimensions (*p* < 0.01). Simultaneously, QoL and its dimensions positively correlated with PR and its dimensions, as well as with SOC and its dimensions (*p* < 0.01). Stigma negatively correlated with PR and its dimensions, as well as with SOC and its dimensions (*p* < 0.01). Additionally, PR and its dimensions positively correlated with SOC and its dimensions (*p* < 0.01).

**Table 3:** Correlations between quality of life, stigma, psychological resilience, and sense of coherence

Variables	QoL	PWB	SWB	EWB	FWB	Stigma	FI	SR	IS	SI	PR	Resilience	Strength	Optimism	SOC	CB	MB	MF
QoL	1.000																	
PWB	0.761^**^	1.000																
SWB	0.621^**^	0.170^**^	1.000															
EWB	0.813^**^	0.677^**^	0.302^**^	1.000														
FWB	0.755^**^	0.411^**^	0.335^**^	0.459^**^	1.000													
Stigma	− 0.647^**^	− 0.577^**^	− 0.302^**^	− 0.610^**^	− 0.430^**^	1.000												
FI	− 0.521^**^	− 0.480^**^	− 0.249^**^	− 0.436^**^	− 0.373^**^	0.710^**^	1.000											
SR	− 0.595^**^	− 0.481^**^	− 0.367^**^	− 0.543^**^	− 0.372^**^	0.858^**^	0.555^**^	1.000										
IS	− 0.402^**^	− 0.365^**^	− 0.176^**^	− 0.422^**^	− 0.234^**^	0.773^**^	0.409^**^	0.503^**^	1.000									
SI	− 0.571^**^	− 0.552^**^	− 0.177^**^	− 0.549^**^	− 0.418^**^	0.877^**^	0.557^**^	0.618^**^	0.604^**^	1.000								
PR	0.597^**^	0.418^**^	0.316^**^	0.514^**^	0.515^**^	− 0.526^**^	− 0.403^**^	− 0.469^**^	− 0.306^**^	− 0.505^**^	1.000							
Resilience	0.533^**^	0.385^**^	0.262^**^	0.455^**^	0.473^**^	− 0.470^**^	− 0.355^**^	− 0.409^**^	− 0.263^**^	− 0.473^**^	0.954^**^	1.000						
Strength	0.591^**^	0.382^**^	0.338^**^	0.524^**^	0.503^**^	− 0.501^**^	− 0.389^**^	− 0.466^**^	− 0.299^**^	− 0.453^**^	0.921^**^	0.785^**^	1.000					
Optimism	0.522^**^	0.394^**^	0.293^**^	0.433^**^	0.421^**^	− 0.495^**^	− 0.387^**^	− 0.436^**^	− 0.306^**^	− 0.464^**^	0.815^**^	0.671^**^	0.753^**^	1.000				
SOC	0.623^**^	0.510^**^	0.250^**^	0.614^**^	0.477^**^	− 0.653^**^	− 0.390^**^	− 0.573^**^	− 0.504^**^	− 0.594^**^	0.581^**^	0.515^**^	0.587^**^	0.499^**^	1.000			
CB	0.573^**^	0.477^**^	0.196^**^	0.613^**^	0.422^**^	− 0.607^**^	− 0.361^**^	− 0.544^**^	− 0.479^**^	− 0.533^**^	0.518^**^	0.458^**^	0.529^**^	0.435^**^	0.924^**^	1.000		
MB	0.524^**^	0.391^**^	0.278^**^	0.463^**^	0.418^**^	− 0.568^**^	− 0.333^**^	− 0.471^**^	− 0.454^**^	− 0.537^**^	0.504^**^	0.437^**^	0.509^**^	0.462^**^	0.881^**^	0.716^**^	1.000	
MF	0.582^**^	0.506^**^	0.202^**^	0.573^**^	0.447^**^	− 0.582^**^	− 0.355^**^	− 0.527^**^	− 0.420^**^	− 0.531^**^	0.547^**^	0.496^**^	0.544^**^	0.449^**^	0.887^**^	0.749^**^	0.659^**^	1.000

*QoL*, quality of life; *PWB*, physiological well-being; *SWB*, social/family well-being; *EWB*, emotional well-being; *FWB*, functional well-being; *FI*, financial insecurity; *SR*, social rejection; *IS*, internalized shame; *SI*, social isolation; *PR*, psychological resilience; *SOC*, sense of coherence; *CB*, comprehensibility; *MB*, manageability; *MF*, meaningfulness; ^*^p < 0.05; ^**^p < 0.01

### Factors influencing QoL among middle-aged and older patients with malignant gynecological tumors

As presented in Table 4, a multiple linear regression analysis was conducted using QoL as the dependent variable and stigma, PR, and SOC as independent variables, while controlling for age, religion, number of children, education level, employment status, per capita monthly household income, family caregiver, presence of concomitant diseases, diagnosis, preoperative adjuvant treatment, and cancer stage. The control variables were dummy-coded before the regression analysis. Collinearity diagnostics indicated no multicollinearity among the independent QoL variables. Sequence correlation analysis revealed no significant correlation within the data, with a Durbin–Watson (DW) value of 1.723. The regression model residuals followed a normal distribution. The results indicated that the regression model had a good fit (*F* = 23.448, *p* < 0.001). The three independent variables (stigma, PR, and SOC) had significant effects on the QoL, explaining 58.7% of the total variation in QoL.

**Table 4:** Multiple linear regression analysis of quality of life

Variables	B	SE	Beta	t	p	VIF	Adjusted *R*^2^	F
Constant	69.576	6.578		10.577	< 0.001^**^		0.587	23.448
Independent variable								
Stigma	− 0.504	0.059	− 0.382	− 8.476	< 0.001^**^	2.099		
Psychological resilience	0.245	0.040	0.255	6.060	< 0.001^**^	1.833		
Sense of coherence	0.183	0.054	0.160	3.418	< 0.001^**^	2.277		
Control variable								
Age (60–74)	− 1.962	0.953	− 0.078	− 2.059	0.040	1.480		
Religion (yes)	1.349	0.912	0.048	1.480	0.140	1.102		
Number of children								
1	2.412	1.908	0.094	1.264	0.207	5.756		
2	1.961	1.945	0.078	1.008	0.314	6.254		
≥ 3	2.827	2.107	0.103	1.342	0.180	6.068		
Education level								
High school or technical secondary school	1.011	1.392	0.026	0.726	0.468	1.306		
Junior college	0.789	2.070	0.014	0.381	0.703	1.366		
Bachelor’s degree and above	0.827	2.442	0.012	0.339	0.735	1.351		
Employment status								
Retired	0.943	1.348	0.030	0.699	0.485	1.839		
Not employed	0.289	1.060	0.012	0.273	0.785	1.908		
Per capita monthly household income								
3000–5999 CNY^1^	− 0.761	0.858	− 0.031	− 0.887	0.376	1.296		
6000–8999 CNY	1.031	1.594	0.023	0.647	0.518	1.258		
≥ 9000 CNY	− 5.513	2.713	− 0.069	− 2.032	0.043	1.202		
Concomitant diseases^2^ (yes)	1.928	0.877	0.077	2.197	0.029	1.258		
Diagnosis								
Endometrial cancer	− 0.406	1.013	− 0.015	− 0.400	0.689	1.427		
Ovarian cancer	− 1.619	1.097	− 0.055	− 1.475	0.141	1.432		
Others^3^	− 3.602	1.709	− 0.069	− 2.108	0.036	1.107		
Preoperative adjuvant treatment (chemotherapy/radiotherapy)	− 1.488	1.570	− 0.033	− 0.948	0.344	1.255		
Family caregiver								
Parents	1.098	2.985	0.012	0.368	0.713	1.170		
Children	− 1.998	1.027	− 0.071	− 1.945	0.053	1.362		
Siblings	− 4.062	2.939	− 0.046	− 1.382	0.168	1.134		
Other relatives	− 3.854	3.719	− 0.034	− 1.036	0.301	1.143		
Cancer stage								
Stage II	− 2.712	1.021	− 0.100	− 2.656	0.008	1.451		
Stage III	− 5.109	1.158	− 0.166	− 4.411	< 0.001	1.460		

*B*, unstandardized coefficient; *SE*, standard error; *Beta*, standardized coefficient; *VIF*, variance inflation factor. All models are adjusted for all listed covariates. ^**^p < 0.01; ^1^CNY Chinese yuan (￥); ^2^concomitant diseases include hypertension, diabetes, asthma, and coronary heart disease; ^3^others include vulvar cancer, vaginal cancer, fallopian tube cancer, choriocarcinoma, uterine sarcoma; reference categories: age = 45–59; religion = no; number of children = none; education level = junior school and below; employment status = employed; per capita monthly household income ≤ 3000 CNY; comorbidity diseases = no; diagnosis = cervical cancer; preoperative adjuvant treatment = no; family caregiver = spouse; cancer stage = stage I

### Measurement model

**Fig. 2:**
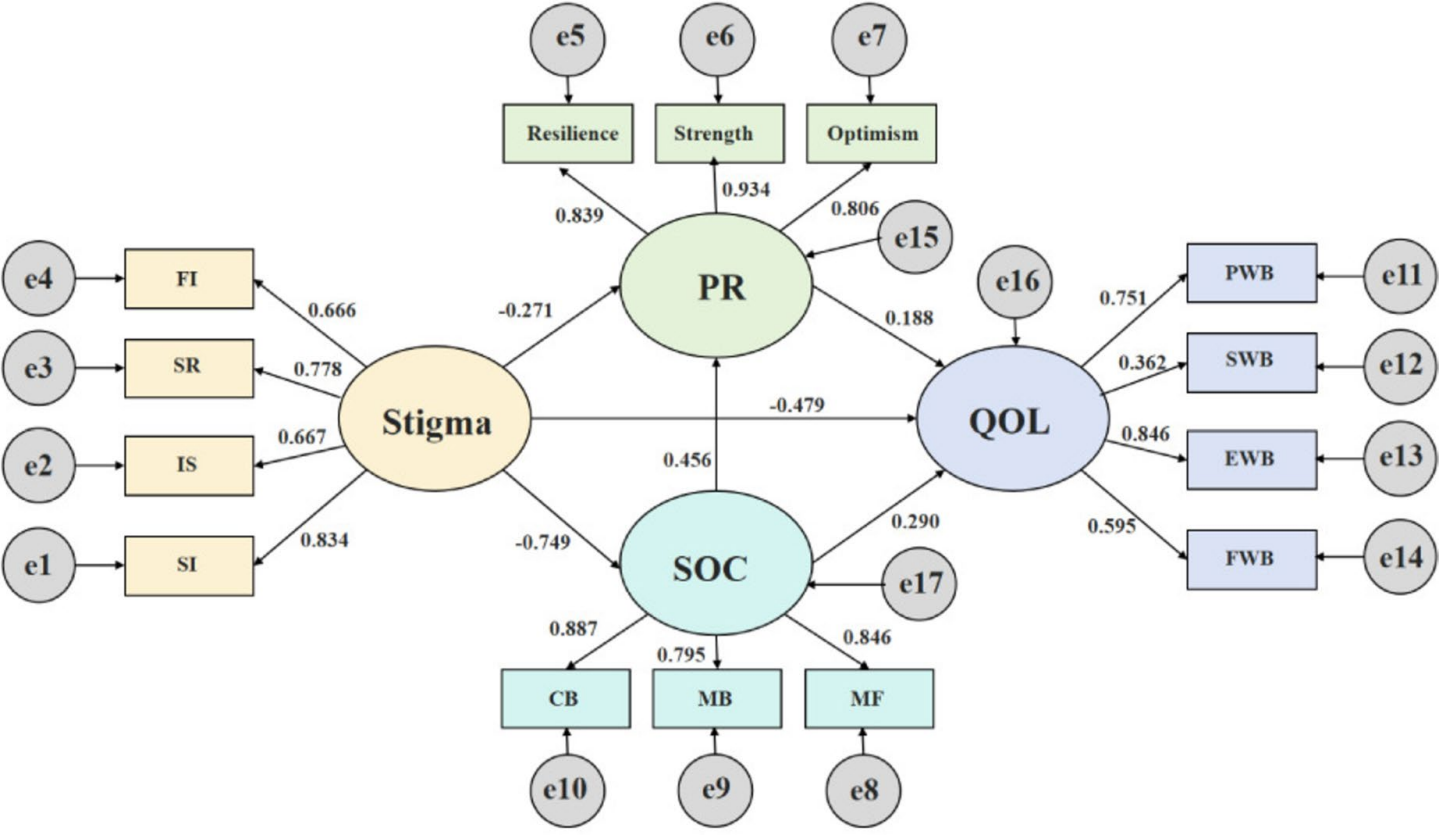
Structural equation modeling results

Figure 2 illustrates the structural equation model, examining the effects of stigma, PR, SOC, and QoL. In this model, QoL was the dependent variable, stigma was the independent variable, and PR and SOC were the mediating variables. The parameters were estimated using maximum likelihood estimation (MLE). The factors leading between the latent and observable variables ranged from 0.362 to 0.934, and the path coefficients ranged from 0.188 to 0.749. All path coefficients in the model were statistically significant (*p* < 0.05). Furthermore, the model exhibited good fit indices, including x^2^/*df* = 3.764, GFI = 0.915, AGFI = 0.874, NFI = 0.925, IFI = 0.944, TLI = 0.928, CFI = 0.943, and RMSEA = 0.080, indicating that it effectively explained the sample data.

Table 5 presents the significance test results for the direct, indirect, and total effects of the mediation model. Stigma had a direct negative effect on SOC (*β* =  − 0.749, 95% confidence interval [− 0.809, − 0.680]). It also had both direct and total negative effects on QoL (− 0.479 [− 0.665, − 0.299] and − 0.811 [− 0.875, − 0.740], respectively). Stigma had an indirect negative effect on QoL through SOC (− 0.242 [− 0.387, − 0.102]). Additionally, stigma had a direct negative effect on PR (− 0.271 [− 0.485, − 0.046]) and an indirect negative effect on QoL through PR (− 0.057 [− 0.146, − 0.006]). Moreover, SOC had a direct positive effect on PR (0.456 [0.260, 0.656]) and both direct and total positive effects on QoL (0.290 [0.116, 0.453] and 0.376 [0.197, 0.541], respectively). Furthermore, SOC had an indirect positive effect on QoL through PR (0.086 [0.025, 0.160]). Stigma had an indirect negative effect on QoL through both SOC and PR (− 0.072 [− 0.138, − 0.021]). Overall, this study demonstrated that SOC and PR partially mediated the relationship between stigma and QoL among postoperative middle-aged and older patients with malignant gynecological tumors.

**Table 5:** Total, direct, and indirect effects of each path in this model

Estimate	β	BC 95% *CI*^‡^		p
		Lower	Upper	
Total effects				
Stigma → QoL	− 0.811	− 0.875	− 0.740	< 0.001^**^
Stigma → SOC	− 0.749	− 0.809	− 0.680	0.001^**^
Stigma → PR	− 0.612	− 0.709	− 0.496	0.001^**^
SOC → PR	0.456	0.260	0.656	< 0.001^**^
SOC → QoL	0.376	0.197	0.541	< 0.001^**^
PR → QoL	0.188	0.033	0.308	0.013^*^
Direct effects				
Stigma → QoL	− 0.479	− 0.665	− 0.299	< 0.001^**^
Stigma → SOC	− 0.749	−0.809	−0.680	0.001^**^
Stigma → PR	− 0.271	−0.485	−0.046	0.022^*^
SOC → PR	0.456	0.260	0.656	< 0.001^**^
SOC → QoL	0.290	0.116	0.453	0.002^**^
PR → QoL	0.188	0.033	0.308	0.013^*^
Indirect effects				
Stigma → PR → QoL	− 0.057	− 0.146	− 0.006	0.021^*^
Stigma → SOC → QoL	− 0.242	− 0.387	− 0.102	0.002^**^
SOC → PR → QoL	0.086	0.025	0.160	0.006^**^
Stigma → SOC → PR → QoL	− 0.072	− 0.138	− 0.021	0.005^**^

*QoL*, quality of life; *SOC*, sense of coherence; *PR*, psychological resilience; ^‡^95% bias-corrected bootstrap confidence interval; ^*^p < 0.05; ^**^p < 0.01

## Discussion

The QoL of cancer patients remains a significant global concern for both the public and researchers. In this study, the mean QoL score among postoperative middle-aged and older patients with malignant gynecological tumors was 60.47 ± 12.03, indicating a poor quality of life. This score is significantly lower than that of patients with metastatic spinal disease [45]. As women age, their physiological and functional status declines, adversely affecting their QoL. Older patients typically experience a lower QoL, consistent with previous research [46]. Additionally, middle-aged and older patients in this study tended to have lower education levels, which may limit their understanding of the disease and exacerbate concerns about prognosis, further reducing QoL [31]. Many of these patients are unemployed, leading to economic instability and reduced ability to manage treatment, which also impacts their QoL [47]. Radical surgeries for gynecological malignancies frequently lead to complications such as bladder dysfunction, gastrointestinal disturbances, and sexual dysfunction, likely attributable to intraoperative pelvic autonomic nerve damage [48]. Perioperative factors such as anesthesia-related symptoms, persistent wound pain, indwelling drainage tubes, and sleep disturbances further degrade QoL [49]. Despite improved survival rates in gynecological oncology, the cumulative burden of treatment side effects underscores the need for targeted QoL interventions.

Among the demographic and clinical factors analyzed, several were associated with QoL, including religion, family caregiver, and cancer stage. Patients with religious beliefs reported higher QoL, consistent with prior research [50]. Notably, patients with spousal caregivers demonstrated significantly better QoL outcomes than those cared for by their children, underscoring the unique value of spousal support in cancer care. Furthermore, advanced cancer stages correlated with lower QoL, likely due to worsening physical and psychological symptoms [51]. Our primary analysis confirmed that stigma directly and negatively affects quality of life, providing robust empirical validation for hypothesis 1. This finding not only aligns with prior evidence demonstrating the deleterious effects of stigma on health outcomes [26, 52], but also extends it. Cancer-related stigma contributes to psychological distress, social withdrawal, and reduced well-being, all of which significantly impair patients’ QoL [18]. Given these detrimental effects, healthcare providers should implement targeted psychosocial interventions aimed at reducing stigma and improving patients’ QoL [53].

Additionally, this study demonstrated that SOC mediated the relationship between stigma and QoL. This mediation effect provided strong support for hypothesis 2, confirming our prediction about the psychosocial mechanism underlying this association. Individuals with a higher SOC were able to use a variety of internal and external resources more effectively to cope with physical and psychological challenges, facing disease and life with a more positive and optimistic attitude, which contributed to a higher QoL [54]. Our study demonstrated that PR mediated the relationship between stigma and QoL, consistent with our previously proposed third hypothesis. The previous study demonstrated that higher Psychological resilience helped mitigate the negative impact of adverse events and improved QoL [55].

The structural equation model analysis in this study revealed that stigma not only directly impacts QoL but also influences it indirectly through SOC and PR. This finding is consistent with Chen’s [56] study and supports hypothesis 4. Stigma, as a pervasive negative psychological phenomenon, can significantly impair patients’ adherence to treatment regimens and their capacity for effective disease self-management. This often results in diminished confidence in the efficacy of medical interventions. Without timely and appropriate intervention, the complexity of managing these conditions escalates, leading to a marked deterioration in patients’ quality of life [57]. Therefore, a comprehensive investigation into the interplay between stigma-induced shame and quality of life among postoperative middle-aged and older patients with malignant gynecological tumors is imperative. Such research is crucial for developing targeted strategies to enhance their overall well-being and treatment. Luo [58] found that in patients with inflammatory bowel disease, stigma significantly impacted their quality of life, mediated by psychological resilience. Resilience has been identified as a significant predictor of self-management in individuals with chronic illnesses. Patients with higher levels of resilience are more inclined to adopt self-management behaviors to better control their conditions. Additionally, various positive personality traits have been shown to mediate the relationship between psychosocial burden and physical health outcomes [59]. Grothe [60] also found that in patients with multiple sclerosis, the sense of coherence emerges as a critical mediator of stigma, presenting a promising target for the refinement of existing stigma interventions aimed at improving the quality of life in affected patients. Those with a strong SOC view the negative effects of illness as challenges rather than threats, perceive these challenges as valuable, and take positive actions to improve their health and QoL. Du Y. W. [61] found that the sense of coherence as an individual’s internal factors affected the formation and development of psychological resilience. The higher the level of individual psychological coherence, the better the development of psychological resilience. Consequently, patients with higher SOC and PR are better able to maintain a positive outlook and manage the pressures of illness, thus mitigating the impact of stigma on their QoL.

### Limitations

This study has several limitations. Firstly, it used convenience sampling, drawing all participants from tertiary hospitals in Fujian Province. Consequently, the sample size may not accurately represent the broader population of middle-aged and older women with gynecological malignancies in China. Future research should aim to include more representative samples from multiple regions. Moreover, the long-term trajectory of QoL in these patients needs further exploration. Understanding the multidimensional factors influencing the QoL of these patients, developing effective interventions, and objective evaluation metrics are essential for improving their QoL.

## Conclusions

This study’s results extend research on the relationship between stigma and QoL. This study shows that stigma is a significant factor in all aspects of the lives of middle-aged and older patients with gynecological malignancies. As hypothesized, the QoL in middle-aged and older patients with gynecological malignancies exhibited a negative correlation with stigma, while exhibiting a positive correlation with SOC and PR. Stigma not only directly impacts QoL but also exerts an indirect effect through the mediating roles of SOC and PR. Therefore, clinical medical staff should focus on reducing stigma and enhancing patients’ SOC and PR to effectively improve their QoL.

## Supplementary Information

Below is the link to the electronic supplementary material.

Supplementary file1 (DOCX 17 KB)

## Data Availability

The datasets used and/or analysed during the current study are available from the corresponding author on reasonable request.
