# Supplementary material for: Mediating effects of sense of coherence and psychological resilience on stigma and quality of life among postoperative middle-aged and older patients with malignant gynecological tumors: a cross-sectional, structural equation model
Source: Support Care Cancer. 2025 Aug 16;33(9):792. doi: 10.1007/s00520-025-09818-7 (PMC12357794; doi:10.1007/s00520-025-09818-7)
Supplement: Supplementary file 1 — Supplementary file1 (DOCX 17 KB) [file 520_2025_9818_MOESM1_ESM.docx]

**Table S1 . Control variable assignment**

| **control variable** | **assignment criteria** |
| --- | --- |
| **Age (years)** | 45-59=0, 60-74=1 |
| **Religion** | NO=0,Yes=1 |
| **Number of children** (Ref: None) |  |
| 1 | 1=1, 2=0, ≥3=0 |
| 2 | 1=0, 2=1, ≥3=0 |
| ≥3 | 1=0, 2=0, ≥3=1 |
| **Education level** (Ref: Junior school and below) |  |
| High school or Technical secondary school | Junior school and below=0, High school or Technical secondary school=1, Junior college=0, Bachelor’s degree and above=0 |
| Junior college | Junior school and below=0, High school or Technical secondary school=0, Junior college=1, Bachelor’s degree and above=0 |
| Bachelor’s degree and above | Junior school and below=0, High school or Technical secondary school=0, Junior college=0, Bachelor’s degree and above=1 |
| **Employment status** (Ref: Employed) |  |
| Not employed | Employed=0, Not employed=1, Retired=0 |
| Retired | Employed=0, Not employed=0, Retired=1 |
| **Per capita monthly household income** (Ref: <3000 CNY^a^) |  |
| 3000–5999 CNY | <3000 CNY =0, 3000–5999 CNY=1, 6000–8999 CNY=0, ≥9000 CNY=0 |
| 6000–8999 CNY | <3000 CNY =0, 3000–5999 CNY=0, 6000–8999 CNY=1, ≥9000 CNY=0 |
| ≥9000 CNY | <3000 CNY =0, 3000–5999 CNY=0, 6000–8999 CNY=0, ≥9000 CNY=1 |
| **Concomitant diseases**^b^ | No=0,Yes=1 |
| **Diagnosis** (Ref: Cervical cancer) |  |
| Endometrial cancer | Cervical cancer=0, Endometrial cancer=1, Ovarian cancer=0, Others=0 |
| Ovarian cancer | Cervical cancer=0, Endometrial cancer=0, Ovarian cancer=1, Others=0 |
| Others^c^ | Cervical cancer=0, Endometrial cancer=0, Ovarian cancer=0, Others=1 |
| **Preoperative adjuvant treatment** | No=0, Chemotherapy / Radiotherapy=1 |
| **Family caregiver** (Ref: Spouse) |  |
| Parents | Spouse=0, Parents=1, Children=0, Siblings=0, Others=0 |
| Children | Spouse=0, Parents=0, Children=1, Siblings=0, Others=0 |
| Siblings | Spouse=0, Parents=0, Children=0, Siblings=1, Others=0 |
| Others relatives | Spouse=0, Parents=0, Children=0, Siblings=0, Others relatives=1 |
| **Cancer stage** (Ref: Stage I) |  |
| Stage II | Stage I=0, Stage II=1, Stage III=0 |
| Stage III | Stage I=0, Stage II=0, Stage III=1 |

Notes: ^a^: CNY Chinese yuan (￥); ^b^: Concomitant diseases include hypertension, diabetes, asthma, and coronary heart disease; ^c^:Others include vulvar cancer, vaginal cancer, fallopian tube cancer, choriocarcinoma, uterine sarcoma；Ref，Reference categories
